# Cellular Responses of Industrially Relevant Silica Dust on Human Glial Cells In Vitro

**DOI:** 10.3390/ijms20020358

**Published:** 2019-01-16

**Authors:** Yke Jildouw Arnoldussen, Torunn Kringlen Ervik, Mina Baarnes Eriksen, Ida Kero, Vidar Skaug, Shanbeh Zienolddiny

**Affiliations:** 1 Department of Biological and Chemical Work Environment, National Institute of Occupational Health, Pb 8149 Dep., N-0033 Oslo, Norway; yke.j.arnoldussen@stami.no (Y.J.A.); torunn.ervik@stami.no (T.K.E.); mina.eriksen@stami.no (M.B.E.); vidar.skaug@stami.no (V.S.); 2 Department of Industrial Process, Technology SINTEF Materials and Chemistry, PB 4760, N-7465 Trondheim, Norway; ida.kero@sintef.no

**Keywords:** amorphous silica, crystalline silica, neurotoxicity, astrocytes, nanotechnology

## Abstract

Despite the rigorous emission control measures in the ferroalloy industry, there are still emissions of dust during the production of various alloys. Dust particles were collected from laboratory scale processes where oxide particulate matter was formed from liquid silicon (metallurgical grade). The dust was produced in a dry air atmosphere to mimic industrial conditions. To investigate possible effects of ultrafine dust on the central nervous system, a human astrocytic cell line was employed to investigate inflammatory effects of particles as astrocytes play a number of active and neuron supporting roles in the brain. Toxicity on the astrocytes by amorphous silica generated in laboratory scale was compared to crystalline macro-sized silica using several doses to determine toxicological dose response curves. The cell viability experiments indicated that low particle doses of amorphous silica induced a small nonsignificant reduction in cell viability compared to crystalline silica which led to increased levels of toxicity. The gene expression of amyloid precursor protein (APP), a biomarker of neurodegenerative disease, was affected by particle exposure. Furthermore, particle exposure, in a dose-and time-dependent manner, affected the ability of the cells to communicate through gap junction channels. In conclusion, in vitro studies using low doses of particles are important to understand mechanisms of toxicity of occupational exposure to silica particles. However, these studies cannot be extrapolated to real exposure scenarios at work place, therefore, controlling and keeping the particle exposure levels low at the work place, would prevent potential negative health effects.

## 1. Introduction

Occupational exposure both to fine and ultrafine silica (SiO_2_) particles present in the dust in the silicon alloy-producing industry has become an area of concern. However, there are continuous efforts to reduce emission levels of Si-containing dust particles [1]. Silica naturally occurs in both crystalline and amorphous forms and because of the extensive natural occurrence of crystalline silica and the widespread use of it in various materials, this increases the possibility of workers to become exposed. The International Agency for Research on Cancer (IARC) has classified crystalline silica as a group I carcinogen [2]. Occupational exposure to crystalline silica is found in industries such as the ceramic industry, cement manufacture, construction, and quartz quarries [3,4,5,6]. In the ferroalloy industry, some crystalline silica dust may be generated during handling and transport of raw materials such as quartz. However, the majority of the airborne particulate matter (PM) in this industry consists of amorphous silica fume. This silica fume is formed in hot processes where liquid silicon is in contact with air. This thermal generation is the main route of formation for fine and ultrafine PM in this industry and is estimated to be the source of up to 85% of the diffuse PM inside a plant [1]. Hence, thermally generated amorphous particles are the most representative and relevant type of silica PM for real, industrial occupational exposure.

Inhalation of the PM leads to partial deposition in the respiratory tract and the alveolar space, where the smallest particles may cross the air-blood-barrier and enter the circulation. The effects of silica exposure to the lung epithelial layer depend on whether the silica is amorphous or crystalline [7]. In addition to the lungs, particles can enter the skin [8] or the olfactory and sensory neuronal pathways, and thereby reach secondary organs such as the brain [9,10]. In the brain, the blood-brain barrier (BBB) protects the brain and crossing and uptake of particles depends highly on the size and level of aggregation, in addition to other physicochemical properties such as the charge and coatings (protein corona) acquired from contact with biological fluids. During recent years where the use of engineered nanoparticles has increased rapidly, silica nanoparticles have become one of the most commonly used nanomaterials in amongst others, biomedical research as a result of several benefits including biocompatibility, stability, and low production costs [11]. As an example, the field of cancer immunotherapy increasingly looks into the use of amorphous silica as immunoadjuvant to treat cancer in humans [12,13,14]. Despite their common and increased use, cytotoxic effects have been reported [15,16]. Due to their biocompatibility in nanomedicine, most molecular and toxicological research has focused on nano-sized silica particles. There is concern on the potential of SiO_2_ to induce inflammatory reactions in glial cells (i.e astrocytes) from the central nervous system (CNS). A study using amorphous SiO_2_ nanoparticles showed that exposure of neuronal cells decreased cell viability, induced oxidative stress, apoptosis, activated p53-mediated stress signaling, and disturbed the cell cycle [17]. It was also shown that SiO_2_ nanoparticles induce apoptosis, oxidative stress, and autophagy in glioblastoma cells [18]. By using a model for the BBB, SiO_2_ nanoparticles induced loss of tight junctions and cytoskeleton arrangement, an increase in the inflammatory response and the release of vascular endothelial growth factor (VEGF) leading to activation of astrocytes [19]. Astrocyte activation occurs in most of the brain pathologies, including neurodegenerative diseases such as Alzheimer’s disease (AD). Activation of astrocytes is associated with changes in the expression of many genes, which, if not resolved in time, can exert inhibitory effects on CNS regeneration [20].

In the present study, amorphous SiO_2_ dust particles were generated and collected from a laboratory scale process. To mimic certain industrial conditions, such as casting, the oxidation process was performed in a dry air atmosphere. Here, active oxidation of liquid silicon surfaces produced very fine spherical particles of amorphous silica and was similar to the essential features of fuming in the industrial oxidative ladle refining of metallurgical grade silicon [21]. Possible toxic effects of the collected dust were investigated in a human astrocytoma cell line. Astrocytes were chosen as they are one of the most abundant non-neuronal cell types in the brain where they have an active role in maintaining numerous processes, such as nervous system repair, regulation of ion concentration in the extracellular space and as support to the BBB. Moreover, astrocytes express amyloid precursor protein (APP), a biomarker of neurodegenerative disease, which after proteolytic processing gives rise to Amyloid β that may accumulate in the extracellular space [22,23]. In this study the cells were exposed to both amorphous (collected in the laboratory process), and crystalline SiO_2_, using a range of low, moderate, and relatively high doses to avoid overload toxicity. A number of cellular endpoints were studied and the findings indicate that cellular responses to SiO_2_ depend on the physicochemical properties of the dust, in addition to the dose and duration of exposure. 

## 2. Results

### 2.1. Characteristics of the Dust

**Figure 1:**
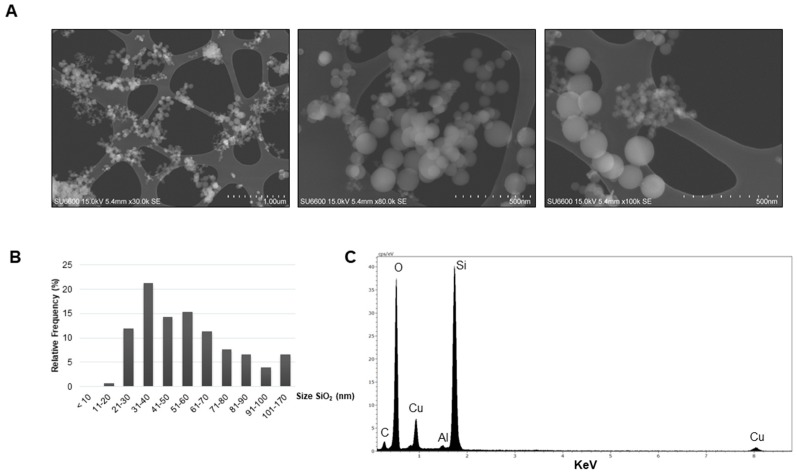

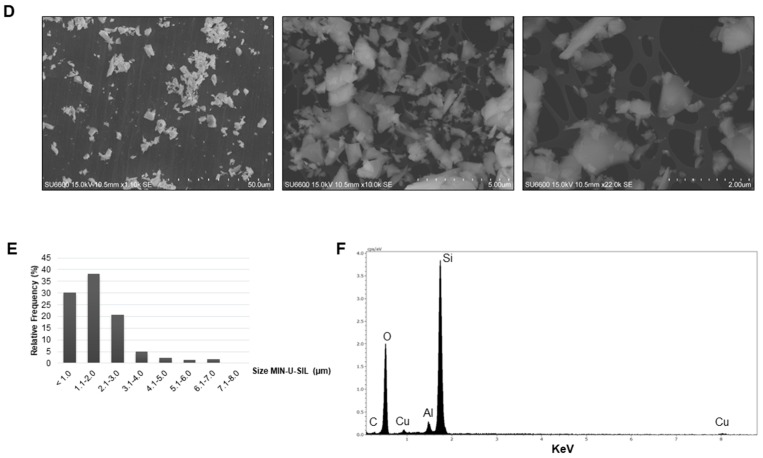
Characterization of the dry dust by scanning electron microscope (SEM). (**A**) Representative SEM images of the amorphous SiO_2_.; (**B**) The diameter (nm) of the dust particles was measured and the relative frequency in percentage is shown for the different size groups (*n* = 300); (**C**) Energy-dispersive X-ray spectrum showing the elemental content of the amorphous SiO_2_ dust; (**D**) Representative SEM images of the crystalline SiO_2_ MIN-U-SIL; (**E**) The diameter (µm) of the MIN-U-SIL dust particles was measured and the relative frequency in percentage is shown for the different size groups (*n* = 300); (**F**) Energy-dispersive X-ray spectrum showing the elemental content of the crystalline SiO_2_ dust.

**Figure 2:**
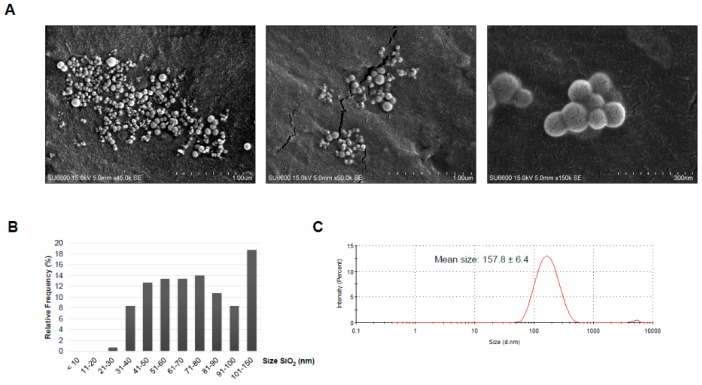

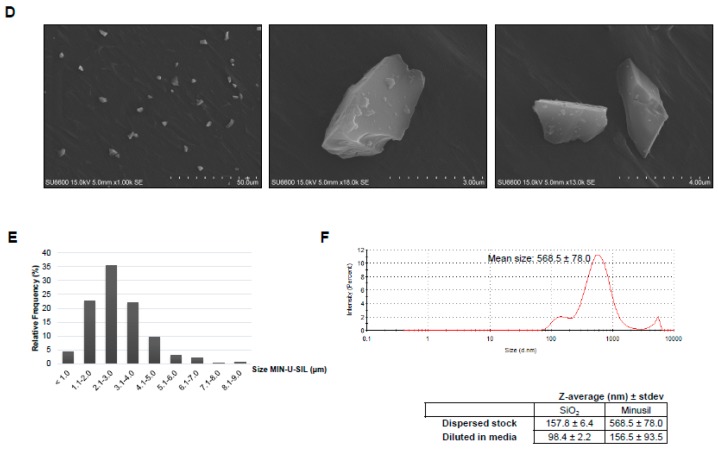
Characterization of the dispersed dust. A volume corresponding to 100 µg dust was taken from a 1 mg/mL stock dispersed in 0.05% BSA and filtered through a 47 mm Whatman Nuclepore polycarbonate filter with 15 nm pore size. The dust was investigated by SEM and representative images are shown for (**A**) amorphous SiO_2_ and (**D**) crystalline SiO_2_; (**B**) The diameter (nm) of the dust particles was measured and the relative frequency in percentage is shown for the different size groups (*n* = 300); (**C**) Size distribution and average hydrodynamic diameter of the dispersed amorphous SiO_2_ dust; (**E**) The diameter (µm) was investigated for crystalline SiO_2_ (*n* = 300); (**F**) Size distribution and average hydrodynamic diameter of the dispersed crystalline SiO_2_ dust. For the dynamic light scattering (DLS) measurements one ml of the dispersed stock solution was to obtain the size distribution and average hydrodynamic diameter. 10 cycles were run and the graphs show the size distribution, which is representative of one measurement over 10 cycles. To sum up the Z-average from three independent dispersed batches is shown ± standard deviation (SD) for both the dispersed stocks and dispersed dust diluted in cell culture media.

Investigation of the dry amorphous SiO_2_ dust by scanning electron microscopy (SEM) showed that it consisted of primary particles of various sizes (Figure 1A). Measurement of the diameter indicated that 93.3% of the primary particles present in the PM were ≤100 nm in size (Figure 1B). The nano-sized particles varied in size with most of the particles being between 31 and 40 nm in diameter (21.3%). The dust consisted of Si and O, as indicated by analysis of the elemental contents. The detected C and Cu are regarded as artefacts from the Cu TEM grid whereas Al probably originates from the sample holder (Figure 1C). The amorphous nature of the silica particles has been described previously by X-ray diffraction (XRD) in Næss et al. [21]. Analysis of dry MIN-U-SIL by SEM showed the presence of larger particles in micron size range (Figure 1D). Measurement of the longest side of the particles indicated particles in the size ranges of <1.0 µm (30.3%), 1.1–2.0 µm (38.3%), and 2.1–3.0 µm (20.7%), in addition to a few of larger size (Figure 1E). None of the MIN-U-SIL dust particles were <100 nm. Elemental analysis showed presence of Si and O, in addition to C and Cu originating from the grid and Al from the sample holder (Figure 1F). The crystalline nature of MIN-U-SIL has been verified previously [24,25]. For cell exposure, the PM was dispersed in solution containing 0.05% *m*/*v* BSA, followed by characterization of the dispersed dust by SEM and dynamic light scattering (DLS). Representative images of the amorphous SiO_2_ and MIN-U-SIL particles in dispersion solution are shown in Figure 2A,D, respectively. Size measurements showed that the primary amorphous SiO_2_ particles were in various nano-size ranges (<100 nm), as well as some above 100 nm (Figure 2B). Measurements of the hydrodynamic size by DLS indicated that the majority of the particles in the solution had a Z-average of 157.8 ± 6.4 nm (Figure 2C). Figure 2D shows three examples of morphologies found by SEM analysis for MIN-U-SIL, and size distribution of the particles is shown in Figure 2E. The largest part of the MIN-U-SIL ranged between 2.1 and 3.0 µm (35.3%) and DLS measurements showed a Z-average of 568.5 ± 78.0 nm (Figure 2F).

### 2.2. The Effect of the Two Types of SiO_2_ Dust on Cellular Endpoints

**Figure 3:**
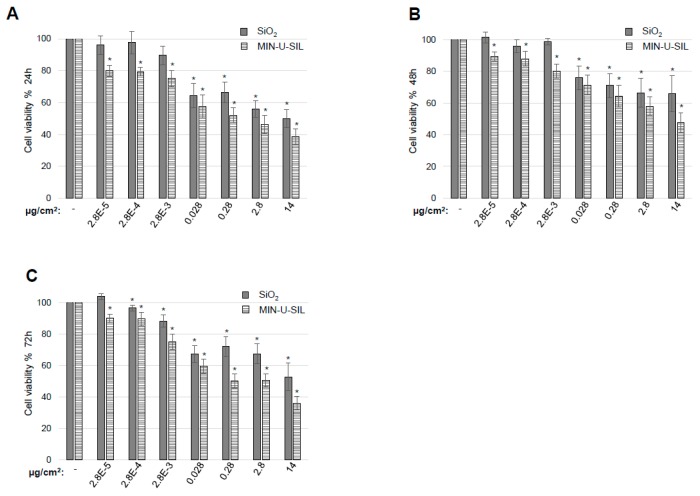
Dust-induced cytotoxicity and gap junctional intercellular communication is dose- and time-dependent. Astrocytoma cells were grown and exposed to control media or to the dust at the indicated concentrations for (**A**) 24, (**B**) 48 and (**C**) 72 h before measurement of cellular cytotoxicity. Cell viability of control-treated cells was set to 100%. An average of three independent experiments in triplicate is shown.

Both types of dust had a dose- and time-dependent effect on cell viability. After 24 (Figure 3A) and 48 h (Figure 3B) of exposure, doses of amorphous SiO_2_ lower than 0.028 µg/cm^2^ did not affect cell viability. However, with the same doses and time of exposure crystalline SiO_2_ induced a significant reduction in viability. Higher doses of both types of dust induced cytotoxicity in the cells. Cells did not recover from the exposure and after 72 h (Figure 3C) there was still a significant reduction in cell viability for both amorphous and crystalline SiO_2_. For all the doses and time points tested it was evident that crystalline SiO_2_ had the largest impact on cell viability compared to amorphous SiO_2_.

**Figure 4:**
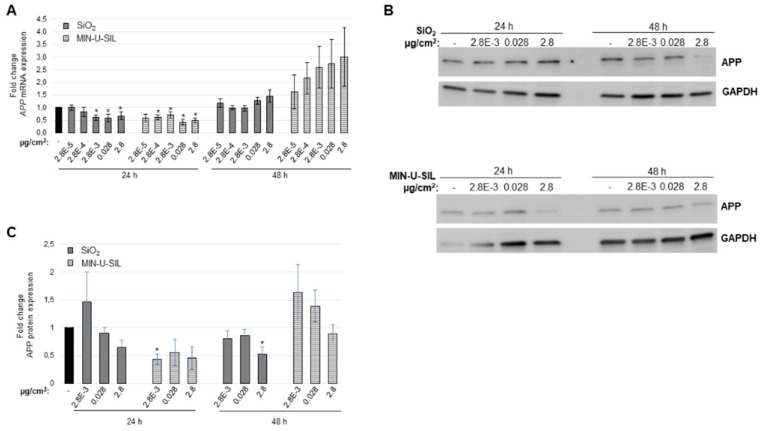
Expression levels of amyloid precursor protein (APP) after exposure to the two types of silica dust. Astrocytoma cells were exposed for 24 and 48 h with the indicated doses. mRNA expression was investigated by qPCR. (**A**) *APP* mRNA expression levels; (**B**) APP protein expression levels. Representative western blot images are shown; (**C**) Quantification of APP expression. An average from three independent experiments is shown. Values represent the mean ± standard error (SE) of two independent experiments performed in triplicate. * *p* < 0.05 between exposed cells and the sham-treated control. Error bars: SE.

To further explore the effect of amorphous and crystalline SiO_2_ particles, the mRNA expression of *amyloid precursor protein* was studied. In addition to neuronal cells in the brain, glial cells such as astrocytes, express APP and may be increased upon cellular stress [22]. Expression analysis of *APP* indicated a significant reduction after 24 h for the three highest doses of amorphous SiO_2_ and for the four highest doses of crystalline SiO_2_ (Figure 4A). After 48 h a non-significant increase is observed with an increasing dose. To investigate if the observations for *APP* mRNA correlated with protein levels, western blot analysis was performed using doses that significantly reduced mRNA expression (Figure 4B). Quantification showed a reduction in APP that was more profound for crystalline SiO_2_ than amorphous SiO_2_ after 24 h, and very similar to *APP* mRNA expression (Figure 4C). After 48 h a decrease in expression was observed with increasing dose and in general higher expression with MIN-U-SIL than amorphous SiO_2_.

**Figure 5:**
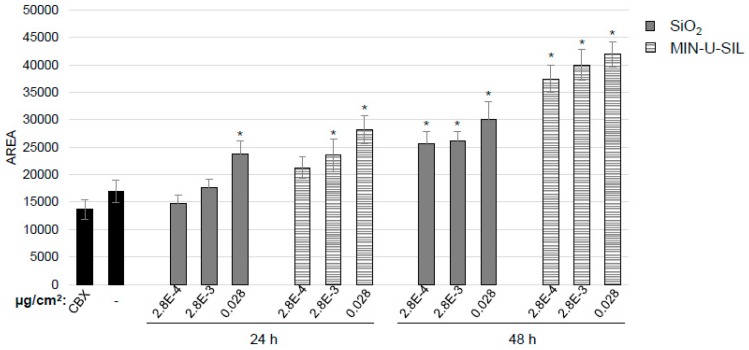
Astrocytoma cells were grown on coverslips and exposed to dispersion media alone as a control or to the indicated doses of dispersed dust for 24 and 48 h. Carbenoxolone (CBX) was included in one well for each experiment as a control for dye uptake solely by the cut in the cell layer. After this time scrape loading was performed using Lucifer Yellow. Confocal microscopy was used to detect fluorescence and the levels of gap junctional intercellular communication were determined by means of the area of dye-coupled cells. Quantification of three independent experiments is shown. Bars: SE. * *p* < 0.05 indicates a significant difference between exposed cells and the corresponding sham-treated control. Error bars: SE.

Astrocytes play an important role in cellular communication in the brain and therefore the level of gap junctional intercellular communication (GJIC) between the cells after exposure to the dust was investigated. For the experiments, the two low, occupationally relevant doses, 2.8 × 10^−4^ and 2.8 × 10^−3^ µg/cm^2^ were used, in addition to 0.028 µg/cm^2^. After 24 h a significant change in GJIC was detected for the highest dose of amorphous SiO_2_, and the two highest doses for crystalline SiO_2_ (Figure 5). After 48 h, all doses used in the experiments induced a significant increase in GJIC (Figure 5). 

## 3. Discussion

This study used dust particles resembling those that employees in the silicon alloy smelters could be exposed to. We found that 93.3% of the dry amorphous SiO_2_ dust consisted of nano-sized particles, in addition to particles in the size range between 100 and 170 nm. However, size determination of this type of dust is affected by several parameters and must be interpreted with some care. First, the silica fume particles are known to agglomerate and aggregate in manners which are more difficult to estimate the size of by SEM [1,26,27]. Hence, the size of the spheres, which in this study is determined visually from SEM images, are representative of the so-called *protoparticle* (primary unit) size but say nothing about agglomerate/aggregate sizes. Characterization of the dispersed dust by SEM also indicated that most particles were in nanometer scale. Secondly, the aggregation of particles may be altered by collection and storage/transport in any container. In addition, sample preparation by ultrasonication or other dispersion methods (in any media) may influence the detected particle size and size distribution. These effects may explain why the particle sizes measured by DLS are larger than with SEM. The sonication treatment in preparation for DLS will have broken most of the weak bonds between agglomerates but not altered the stronger bonds between aggregates, thus the DLS results describe the hydrated aggregate sizes. It is important to emphasize that the present study used an industrially relevant, polydisperse type of SiO_2_ dust which is in contrast to other studies that mainly use monodisperse, amorphous SiO_2_ nanoparticles to investigate potential health effects. However, the aggregation state of the real, industrial dust in its airborne state has not been described, evaluated, or reproduced in this study. Industrial silica fume has been detected and characterized by a number of authors and the presence of fine and ultrafine particles (in the airborne state) has been documented [1,21,26,27,28,29].

In this study astrocyte derived astrocytoma cells were used to investigate possible cellular effects. In the brain, astrocytes play an important role in maintaining the BBB and help to separate the brain from the systemic circulation. It has been shown that nanoparticles may translocate across the BBB [30], but there is not much data on the actual amount of particles that may translocate to the brain, and whether it is through the olfactory nerves or via the pulmonary system.

Studies on cellular effects of silica in glial cells of the brain are very limited (reviewed in [31]). Compared to other studies, low doses of respirable silica dust were used in this study, but it is likely that the translocated amount into the brain is even lower than the doses used here. In general, the toxicity of silica nanoparticles depends on their size, dose, duration of exposure and the cell type used in in vitro studies. Also, amorphous SiO_2_ was shown to induce toxicity through mechanisms similar to those for crystalline SiO_2_. Studies on the rat neuronal cell line PC12, showed that direct exposure to 25 nm amorphous SiO_2_ nanoparticles with doses ranging from 25 to 200 µg/mL led to uptake of the particles and a dose-dependent induction of the cell death mechanism of autophagy [32]. In the present study, cell viability was only significantly reduced by doses that may not be relevant to occupational exposure settings. The slight increase in viability after 48 h indicates that cell proliferation and survival of the cells increased. For the lower doses cell viability was affected in a smaller degree. There was a clear difference in the reduction in cell viability induced by amorphous SiO_2_ compared to crystalline SiO_2_. Crystalline SiO_2_ is known to have toxic effects on several cell lines in vitro and animal studies in vivo [33,34,35] that with the present study also are confirmed in an astrocytic cell line. We cannot exclude that there can be traces of Al in the silica dust or in the MIN-U-SIL, however, in that case these would be only trace amounts of Al and should not have large effects on the toxicity of the SiO_2_ particles.

Evaluation of a marker for neurotoxicity, APP, a precursor for Amyloid β that accumulates extracellularly in neurodegenerative diseases such as Alzheimer’s, indicated dose-and time dependent effects of the two types of dust. Amyloid β and astrocytes play an important role in Amyloid β-related disorders and an increase in Amyloid β is associated with chronic inflammatory responses in the brain [36]. A study in neuroblastoma cells showed an increase in Amyloid β expression after exposure to amorphous SiO_2_ nanoparticles, which also reduced cell viability and increased apoptosis [37]. In our study, a significant downregulation in mRNA expression was observed with the higher doses after 24 h that was reversed after 48 h. Thus, the initial decrease in mRNA expression may be part of a survival mechanism and initial cellular response, but as the dust particles’ effect is too severe, as shown by the reduction in cell viability, the expression of *APP* increases after 48 h. Conversely, a reduction in APP protein expression was observed with increasing dose. As APP undergoes cleavage giving rise to Amyloid β, this may indicate that SiO_2_, in particular crystalline SiO_2_, positively affects cleavage of APP leading to lower levels of the precursor protein. A similar observation was done in neuroblastoma cells where UV irradiation increased *APP* mRNA and simultaneously decreased APP protein levels [38].

In parallel to the reduction in cell viability, there was a dose-and time-dependent effect on GJIC. The level of GJIC increased and was affected in a higher degree by crystalline SiO_2_ than the amorphous SiO_2_. GJIC is an important feature of astrocytes and is involved in resistance to oxidative stress [39,40]. Previous studies have, however, shown that the 1321N1 cell line displays low intercellular communication as indicated by low dye coupling and low connexin-43 levels [41,42]. This was also confirmed in the present study where in general the amount of GJIC was low as demonstrated by the non-significant effect of the GJIC-inhibitor carbenoloxone. Despite this, GJIC increased with increasing time and dose in parallel to a reduction in cell viability and may therefore indicate that there is an increase in anti-survival signals between the cells. These data also correspond to results obtained in the same cell line exposed to a different kind of dust [43].

The actual amount of particles that may pass from the lungs to the blood and eventually reach the brain is currently unknown as studies are inconclusive. This will also be affected by differences in breathing, how much is deposited in the upper airways and the amount of particles that are cleared from the airways. Therefore, the actual doses that possibly reach the brain may be even lower than the ones used in this study. Furthermore, the astrocytoma cell line 1321N1 used in this study is a transformed cell line, and it would therefore be of interest to perform the same type of study on primary astrocyte cells. However, as the 1321N1 cell line is a well-established cell line in neurotoxicology research, it is a good model for providing initial information on possible effects of silica exposure.

In summary, the laboratory-produced SiO_2_ dust contained mostly nano-sized particles. The effects of this amorphous dust were compared to those of micron-sized crystalline SiO_2_. Both types of dust had a dose-and time-dependent effect on the astrocytoma cells where high dust doses induced the most significant changes. Moreover, the crystalline SiO_2_ affected the cells more severely than the amorphous SiO_2_. In addition, intercellular communication and mRNA expression of *APP* increased with increasing dose and time, in addition to a reduction in APP. This in vitro study may contribute to understand the biological mechanisms of silica at the cellular level and cannot be extrapolated to real exposure scenarios at the work place. Furthermore, it should be noted that it has not been taken into account how many percent of the particles deposited in the alveolar space may translocate to the circulation and may reach the blood brain barrier before reaching the astrocytes. In conclusion, even though no significant toxic effects were observed at low doses, the appropriate protective measures at work place should be applied in order to avoid any potential health effects.

## 4. Materials and Methods

### 4.1. Generation of the Dust

The amorphous silica fume was generated in an alumina tube furnace, with an inner diameter of 5.2 cm. The general experimental set-up has been described in detail in previous publications [21]. For these studies, a sample of approximately 10 g photovoltaic grade silicon (9 N) was melted and kept at 1500 °C with a gas flow of argon and oxygen (1 vol.% O_2_; laminar flow rate 0.047 m/s). The particulate matter used in these studies were collected from the alumina tube, near (1–15 cm from) the gas outlet. MIN-U-SIL (crystalline SiO_2_) was kindly provided by Dr. Magne Refsnes (Norwegian Institute of Public Health, Oslo, Norway), and was initially produced by U.S. Silica.

### 4.2. Preparation of the Dust for Characterization and Cell Culture Experiments

For dispersion of the dust, the NANOGENOTOX protocol for dispersion of nanomaterials was used with small adjustments [44,45]. Briefly, the dust was weighed and a solution of sterile-filtered 0.05% Bovine Serum Albumin (BSA; *m*/*v* in H_2_O) was added to obtain a stock solution of 1 mg/mL. BSA was used to get a well-dispersed dust. After a brief vortexing, the solution was sonicated using a probe sonicator at 10% amplitude (Sonifier 450S, Branson Ultrasonics, Danbury, CT, USA) for 15 min. For each single experiment, a freshly prepared stock was used. 

### 4.3. Dust Characterization

#### 4.3.1. Scanning Electron Microscopy (SEM)

Both dry and dispersed dust in solution were analyzed. Dry dust was prepared as follows: A Copper (Cu) TEM grid with holey carbon film (Holey Carbon film on Copper H7, EM Resolutions Ltd., Sheffield, UK) was fixed onto a 25 mm polycarbonate (PC) filter with a pore size of 1 µm. The particles were spread on an aluminum plate and collected on the Cu TEM grid using open-face graphite-filled 25 mm filter holders with 50 mm extension tube (Gelman Air Monitoring Cassette, Gelman Sciences, Ann Arbor, MI, USA) through a 2 L/min nozzle. The particles on the Cu TEM grid were investigated without any further preparation. For characterization of dispersed dust, a volume corresponding to 100 µg was taken from a 1 mg/mL stock dispersed in 0.05% BSA which was sonicated as described above followed by filtering on a 47 mm Whatman Nuclepore polycarbonate filter with 15 nm pore size. Thereafter the filter was coated with a thin platinum film in a sputter coater (Cressington 208HR sputter coater, Watford, UK). Specimens of 10 × 10 mm were cut from the filter and gently fixed on aluminum specimen stubs with double-sided carbon adhesive discs. All specimens were analyzed with a Hitachi SU 6600 (Ibaraki-ken, Japan) field emission scanning electron microscope (FE-SEM) equipped with a Bruker energy-dispersive X-ray (EDX) detector (Bruker Nano GmbH, Berlin, Germany). The instrument was operated under the following conditions during EDX elemental analysis: accelerating voltage 15 keV and working distance 10 mm. High resolution images of the particles were obtained by acquiring at slow scanning speed. Initially specimens were examined in the SEM to determine their morphology and size.

#### 4.3.2. Dynamic Light Scattering (DLS)

To obtain information on the dusts’ hydrodynamic size distribution after dispersion, the ZetaSizer Nano ZS (Malvern Instruments Ltd., Malvern, UK) was used. Immediately after sonication 1 mL of the sonicated solution was pipetted into a cuvette, left on the bench for 5 min and was thereafter left for five minutes in the ZetaSizer apparatus before measuring over 10 cycles. ZetaSizer software (Malvern Instruments Ltd., Malvern, UK) was used to analyze the data. The results shown are from three independent measurements. Similarly, the dusts’ size distribution was measured after dispersion in cell culture media. 1 mL of cell culture media containing the highest concentration of dust particles for the experiments was added to the cuvette and measured using ZetaSizer.

### 4.4. Cells and Cell Culture

The human astrocytoma 1321N1 cell line was purchased from Sigma-Aldrich (St. Louis, MO, USA; catalogue no. 86030402). These are glial cells from a human brain astrocytoma that was initially isolated in 1972 as a sub clone of the cell line 1181N1 [46]. Cells were routinely kept in a humidified 5% CO_2_ and 95% air incubator at 37 °C in Dulbecco’s Modified Eagle’s Medium (DMEM, Fisher Scientific, Hampton, NH, USA) containing 10% fetal bovine serum (FBS, Biochrom, Cambridge, UK), 50 U/mL penicillin and 50 μg/mL streptomycin (Thermo Scientific, Waltham, MA, USA). The passage number of the cells was kept below 30.

### 4.5. Estimation of Dust Doses Used for the Experiments

The doses used for cell culture exposures were calculated following a mathematical calculation modified from Antonini and coworkers [47,48] to determine the daily lung burden of a worker working 8 h per day. Incorporated factors were the occupational exposure limit for the respirable fraction of amorphous silica dust (1.5 mg/m^3^), human minute ventilation volume (20.000 mL/min × 10^−6^ m^3^/mL), the exposure duration (8 h/day), and the deposition efficiency (set to 20%; [49,50]). 

The daily deposited lung dose was calculated as follows:

1.5 mg/m^3^ × (20.000 mL/min × 10^−6^ m^3^/mL) × (8 h × 60 min/h) × 0.20 = 2.88 mg

When using the surface area of the alveolar epithelium (human 102 m^2^ [51]) this leads to a deposited dose of 9 × 10^−4^ µg/cm^2^ (2.88 mg/1.020.000 cm^2^ = 2.8 × 10^−6^ mg/cm^2^ → 0.0028 µg/cm^2^). This dose was set as 1× and the other doses (0×, 0.01×, 0.1×, 1×, 10×, 100×, 1000×, 5000×) used in this study were calculated accordingly. Thus, cells were exposed to 0, 2.8 × 10^−5^, 2.8 × 10^−4^, 2.8 × 10^−3^, 0.028, 0.28, 2.8 and 14 µg/cm^2^ taking into account the surface area of the cell culture dish, respectively. For the use of MIN-U-SIL as a positive control throughout the experiments, the same doses as for amorphous silica were used. In this calculation it has neither been taken into account the percentage of particles crossing the air-blood barrier nor the blood-brain barrier.

### 4.6. Cytotoxicity Assay

For each toxicity experiment 5000 cells/well were seeded in triplicate in black 96-well plates with a transparent bottom (Nunclon, Thermo Scientific, Waltham, MA, USA). Cells were allowed to attach for 24 h prior to addition of dispersed amorphous SiO_2_ dust or MIN-U-SIL at the indicated doses. Thereafter the medium was removed and the cells were washed once with PBS to remove excess material. The Cell Counting Kit-8 (CCK-8) assay (Sigma-Aldrich, St. Louis, MO, USA) was used to measure cytotoxicity levels by diluting it in the cell culture media without supplements according to the manufacturer’s instructions. After incubation at 37 °C for 1 h, absorbance as optical density (OD) was measured at 450 nm using a SpectraMax i3 (Molecular Devices, San Jose, CA, USA). In addition, OD was measured at 750 nm as a reference wavelength for background detection and was subtracted from sample OD at 450 nm. A standard curve with a known number of cells was established to calculate the number of cells in each well. 

### 4.7. Quantitative PCR (qPCR) for Measurement of Gene Expression

The mRNA levels of the neurodegenerative disease marker *APP* was measured by qPCR. Briefly, total RNA was extracted using RNA-Solv reagent (OMEGA bio-tek, Norcross, Georgia, USA). cDNA from one µg of RNA was prepared using qScript cDNA synthesis kit (Quanta Biosciences, Beverly, MA, USA) according to the manufacturers’ recommendations. qPCR was performed on a StepOne Real-Time PCR system (Applied Biosystems, Foster By, CA, USA) with Perfecta SYBR Green FastMix, ROX (Quanta BioSciences, Beverly, MA, USA). Pre-designed primers were purchased from Sigma-Aldrich (St. Louis, MO, USA). A serial diluted internal standard served as a control for the qPCR reaction. Relative gene expression levels were calculated and normalized to the average expression levels of *β-actin*, *GAPDH* and *TBP*.

### 4.8. Detection of APP by Western Blotting Analysis 

Western blot analysis was performed as described previously [52]. Briefly, protein concentrations were measured using NanoDrop-8000 (Thermo Scientific, Waltham, MA, USA). A total of 25 µg of each protein sample was resolved on AnykD Mini Protean TGX stain free gels (Bio-Rad Laboratories, Hercules, CA, USA) and transferred to a PVDF membrane (Bio-Rad Laboratories, Hercules, CA, USA). The Trans-Blot Turbo blotting system (Bio-Rad Laboratories, Hercules, CA, USA) was used for transfer. Antibodies used were as follows; APP (Cell Signaling Technology, Danvers, MA, USA) and GAPDH (Santa Cruz Biotechnology, Dallas, TX, USA). Horseradish peroxidase conjugated antibodies (Cell Signaling Technology) were used prior to chemiluminescent detection (GE Healthcare, Chicago, IL, USA). Results were quantified using Image J.

### 4.9. Functional Assay of Gap Junctional Intercellular Communication (GJIC) by Scrape Loading

GJIC was determined by quantitative scrape loading [53]. 1321N1 cells were cultured on cover slips in 12-well plates (NUNC) and grown until 80–90% confluency. The cells were then exposed to the indicated doses of the dust for 24 and 48 h. Before scrape loading, the confluent cell layer was washed twice with PBS. Then 1 mL of 0.05% Lucifer Yellow (Sigma-Aldrich, St. Louis, MO, USA) dissolved in PBS w/o Ca^2+^ and Mg^2+^ was added to each well and the cell monolayer was cut with a surgical scalpel four times. After 4 min the Lucifer Yellow solution was removed, the well was washed with PBS four times and then cells were fixed in 3.7% formalin overnight. The next day the wells were washed with PBS two times before mounting of the cover slips with Mowiol. During the whole experiment, cells from one well were exposed to the gap junctional inhibitor carbenoxolone (CBX) (100 µM, Alfa Aesar, Haverhill, MA, USA) as a control for dye uptake solely by cutting the cell layer. Fluorescence was observed using a laser scanning microscope (LSM 710, Zeiss, Oberkochen, Germany) with a magnification of 20× and photographs were taken with an AxioCam camera (Zeiss, Oberkochen, Germany). Ten images were taken for each exposure. Analysis was done by the public domain NIH Image program. The same settings were used for each measurement. The levels of GJIC were determined as the distance of diffusion of the dye away from the scalpel cut. The average area of diffusion obtained for the CBX exposed control cells was included in the figures.
